# Proceeding American Institute of Dental Teachers

**Published:** 1914-06-15

**Authors:** 


PROCEEDING AMERICAN INSTITUTE OF DENTAL TEACHERS. The transactions of the Institute of Dental Pedagogics at Buffalo, in January have just been issued in pamphlet form as usual and are published by the Institute rather than through a dental journal. These transactions are valuable as they represent the teaching material and progress in dental instruction for the country. The papers are of so much interest that it seems as though they should be given a wider reading through one of the more largely circulated dental journals. This seems difficult of accomplishment because of the fact that the teachers are anxious to get the matter as soon as possible and the journals spread it out over several issues which consumes time. The change in name of this organization seems to be unnecessary, but many of the members think it will be more popular with a new name. It is however the same organization in fact that has prospered under its three names.

